# Illusory Changes in Body Size Modulate Body Satisfaction in a Way That Is Related to Non-Clinical Eating Disorder Psychopathology

**DOI:** 10.1371/journal.pone.0085773

**Published:** 2014-01-21

**Authors:** Catherine Preston, H. Henrik Ehrsson

**Affiliations:** Brain, Body and Self Laboratory, Department of Neuroscience, Karolinska Institutet, Stockholm, Sweden; University G. d'Annunzio, Italy

## Abstract

Historically, body size overestimation has been linked to abnormal levels of body dissatisfaction found in eating disorders. However, recently this relationship has been called into question. Indeed, despite a link between how we perceive and how we feel about our body seeming intuitive, until now lack of an experimental method to manipulate body size has meant that a causal link, even in healthy participants, has remained elusive. Recent developments in body perception research demonstrate that the perceptual experience of the body can be readily manipulated using multisensory illusions. The current study exploits such illusions to modulate perceived body size in an attempt to influence body satisfaction. Participants were presented with stereoscopic video images of slimmer and wider mannequin bodies viewed through head-mounted displays from first person perspective. Illusory ownership was induced by synchronously stroking the seen mannequin body with the unseen real body. Pre and post-illusion affective and perceptual measures captured changes in perceived body size and body satisfaction. Illusory ownership of a slimmer body resulted in participants perceiving their actual body as slimmer and giving higher ratings of body satisfaction demonstrating a direct link between perceptual and affective body representations. Change in body satisfaction following illusory ownership of a wider body, however, was related to degree of (non-clinical) eating disorder psychopathology, which can be linked to fluctuating body representations found in clinical samples. The results suggest that body perception is linked to body satisfaction and may be of importance for eating disorder symptomology.

## Introduction

Eating disorders (EDs), such as anorexia nervosa (AN) and bulimia nervosa (BN), are serious psychiatric conditions primarily afflicting young women. Compared to other disorders EDs have high relapse [1] and mortality [2] rates as well as prevalence of long-term physical [3] and psychological problems [4]. An important element thought to contribute to the development, maintenance and relapse of EDs is high levels of body dissatisfaction [5]. Therefore, improving the affective experience of the body has been identified as a key target for preventative and therapeutic intervention [6].

Historically, one aspect thought to contribute to body dissatisfaction in EDs, particularly for AN, is an overestimation of body size, with abnormal body processing found in ED patients using a number of methods; from size judgments using self photographs [7] and callipers [8], to tactile estimation [9] and body scaled action [10], [11]. However, the simplicity of this relationship has been challenged, suggesting that AN body misperception is no different from underweight controls [12] or at least not a universal trait for all patients [13]. Another possibility is that ED (AN and BN) body representations are unstable and fluctuate depending on the environment, which may account for inconsistent results [14]. However, despite the volume of studies with EDs and those relating body mass index (BMI) to body dissatisfaction in healthy participants (e.g. [15]–[18]), the lack of a successful empirical method to modulate body size means that a direct causal link to body satisfaction has remained elusive for either group.

Recently, growing interest in the scientific study of body perception and body ownership has led to the development of various different multisensory body illusions. The classical of these, the rubber hand illusion (RHI), induces feelings of ownership over a fake hand by touching the seen fake hand in synchrony with touch delivered to the hidden real hand [19]. Touching the hands asynchronously, however, abolishes the effect. Petkova and Ehrsson [20] expanded this concept, demonstrating that viewing a mannequin body from first person perspective, whilst also experiencing synchronous visual-tactile stimulation induces illusory ownership over the fake body. Such illusions are demonstrated by subjective reports of ownership, but also with objective measures. For example, attacking the ‘owned’ (synchronously stroked) body (part) with a threatening object (e.g. a knife or a hammer) can cause heightened skin conductance as if the real body was being threatened [21]–[22]. Modulation of these illusions can also produce perceptual changes to the shape and size of the actual body, for example, illusory elongation of a finger [23] or arm [24], [25] and illusory expansion of the stomach [26]. However, despite causing profound changes to how we perceive our body, to date the effect of these illusions on how we feel about our body (affective body representation) remains unknown.

The current study will be the first to exploit these well-established illusions to investigate a causal link between body perception and body satisfaction over two separate experiments. The full body illusion will be induced over a mannequin body digitally manipulated to be both wider and slimmer than the participants’ actual body size. Experiment one seeks to establish equivalent perceptual effects for both body types using subjective reports and objective skin conductance to threatening stimuli with male and female participants. Using male mannequins males and females do not differ in terms of perceptual effects of multisensory illusions [20]. However, due to the importance of gender specific body features to ideals of body appearance the current study will use sex-matched mannequins, with which it is predicted that male and female participants will also report equally strong illusions for both body sizes.

Experiment two then aims to measure affective responses by comparing pre and post-illusion measures of body satisfaction. It is hypothesised that significant increases in body satisfaction will occur following illusory ownership over a slimmer body, whereas a larger body will decrease body satisfaction. Because females generally have lower body satisfaction that is related more strongly to body size (e.g. [15], [16]), it is also predicted that females will have enhanced emotional responses to equivalent perceptual effects.

Moreover, to investigate the relationship with EDs, levels of ED psychopathology will be recorded for each participant. Due to recent evidence associating EDs with more readily fluctuating body representations [14] it is predicted that higher (non-clinical) scores will correspond with greater affective and perceptual effects of the illusions.

## Methods

### Experiment 1

Experiment one aimed to determine equivalent illusion strength for male and female participants with both wide and slim body sizes.

#### Participants

38 participants (19 male) with a mean age of 25 years (range 19–42) took part in the study. Males had a mean age of 28 years and females 23 years. All participants gave written informed consent and both experiments were conducted in accordance with the declaration of Helsinki and were approved by the Swedish Central Ethical Review Board.

#### Materials

During the experiment, participants wore a set of head-mounted displays (HMDs) (Cybermind Visette45, Cybermind Interactive, Maastricht, the Netherlands) with a field of view of 45° and a display resolution of 1280×1024. The HMDs were connected to two synchronized colour Stingray F-125C cameras (Allied Vision Technologies), which were mounted on a tripod that stood on a slanted tabletop (∼40°) positioned to record the mannequin’s body from above (first person perspective). The cameras were attached side-by-side with the centre of the lenses 7 cm apart (within normal range of human interpupillary distance; [27]). The images from the left and right video cameras were projected onto the left and right side of the HMDs respectively, producing a true stereoscopic image.

A life-size male mannequin was used for male participants and a female mannequin for female participants. For the original full body illusion, naked mannequins were used [20]. However, to prevent emotional reactions associated with not wearing clothes each mannequin was dressed in a white t-shirt, blue jeans, and trainers [28]. The clothing also served to hide muscle definition on the male torso. The mannequin's head was removed at the neckline to enable correct positioning of the video cameras.

Skin conductance response (SCR) was recorded using a pre-established protocol [20]: Biopac System MP150 (Goleta, USA), with the gain switch set to 5 mmho/V and a CAL2 Scale Value of 5. Electrodes were attached to the index and middle fingers of the left hand using Signa electrode gel (Parker Laboratories, INC., New Jersey, USA). Skin conductance, as a measure of autonomic arousal, was recorded at 100 samples per second and processed with the Biopac software package Acknowledge for Windows (ACK100W). A 33 cm kitchen knife (21 cm blade) was used to threaten the mannequin. For analysis, SCR was defined as the peak amplitude within five seconds after onset of the knife threat and was calculated by identifying the maximum conductance value and then subtracting the preceding minimum value within the time frame. The onset of the knife threat was recorded in the data files by the experimenter pressing a key when the knife entered the image. SCR non-responders (N = 10) were excluded from analysis based on criteria used by [29].

#### Procedure

**Figure 1:**
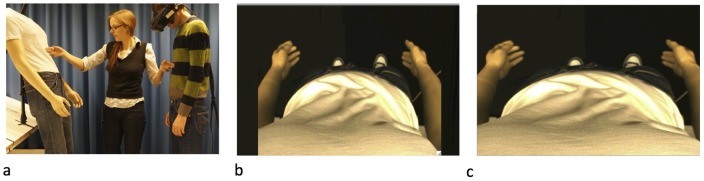
Experimental set- up. a) The illusion was induced by synchronously stroking the mannequin body and the corresponding part of the participant’s body. Participant view through the head mounted display of the male mannequin in the small (b) and large (c) body conditions. Both subjects in the figure have given written informed consent, as outlined in the PLOS consent form, to publication of their photograph.

The experiment utilised a within participants design. Participants stood facing the mannequin wearing the HMDs with their head tilted forwards as if looking down at their own body. An image of the mannequin's body viewed from first person perspective was presented through the HMDs therefore appearing in place of their real body (see Figure 1). For each trial the experimenter stroked both the mannequin and the actual torso for 60 seconds. Stroking was divided equally between the centre, left, and right side of the torso.

For synchronous trials the experimenter stroked the mannequin torso and the corresponding position on the participant's torso in complete temporal synchrony. In asynchronous trials the touch was delivered to the torsos at different times (∼1 s delay). The stroking pattern was kept irregular to avoid predictability effects.

There were two body size conditions: large body (LB) and small body (SB). Body size was measured relative to the outer edges of the body at the hipbones and the distance of the participants actual hip measurement was compared to that of the mannequin so that the digital adjustments applied were 115% and 85% of the participants own body size for LB and SB conditions respectively. Both body size conditions were completed with synchronous and asynchronous stroking making a total of four conditions: LB synchronous, LB asynchronous, SB synchronous and SB asynchronous.

Out of a total of 16 trials, the first 12 (three per condition) measured skin conductance response to a knife threat as an objective measure of the illusion. Following 60 s of stroking the experimenter attacked the mannequin with a knife whilst measuring SCR. The knife approached the mannequin torso from the left and was drawn across the body from left to right being in view for approximately two seconds and in contact with the mannequin for approximately one second. These trials were conducted in a random order with a minimum of one two-minute break every four trials in which participants removed the HMDs.

**Figure 2:**
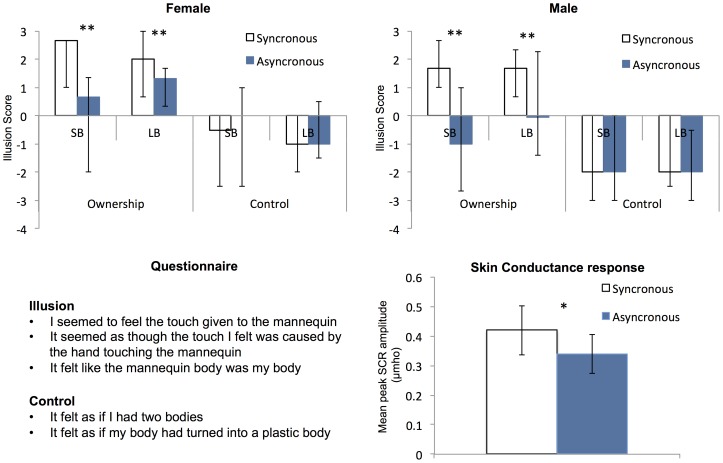
Results of Experiment one. Median illusion and control questionnaire scores for both female (a) and male (b) participants. Greater agreement was found following synchronous (open bars) compared to asynchronous (filled bars) stroking for the illusion but not control questions. Error bars show interquartile range. c) Questionnaire items used to calculate control and illusion scores. Participants gave numeric responses from +3 (strongly agree) to −3 (strongly disagree). d) Mean skin conductance response (SCR) to the knife threatening the mannequin. Greater amplitudes were found following synchronous stroking. Error bars show standard errors. * = p<.05, ** = p<.01.

In the final four trials (one per condition), following 60 s of stroking, participants rated their experience using a seven-point Likert scale, +3 (strongly agree) to −3 (strongly disagree). The questionnaire consisted of seven statements, three aimed at the strength of the illusion and two control questions (based on [19] see Figure 2), with an additional two questions asking directly about perceived body size (see below). All questionnaire items were presented to the participant in a random order via the HMDs with (numeric) responses given verbally to the experimenter. Breaks of at least two minutes in which the participant removed the HMDs were given between each trial. The duration of the entire experiment was approximately 50 minutes. Subjective responses were taken at the end of the experiment to keep participants naïve as to the purpose of the study for as long as possible. Similarly, subjective responses were only taken once per condition to prevent participants from guessing the aim of the experiment and thus reducing the possibility of demand characteristics. This approach has been used by previous similar studies (e.g. [20]).

### Experiment 2

Experiment two aimed to investigate affective responses to illusory ownership over both LB and SB body sizes using a new cohort of participants.

#### Participants

To control for possible confounding variables that may effect body satisfaction, additional measures and screening were completed for this sample (see table 1, table S2, and below).

**Table 1:** totals and statistics for full sample and males and females for age body mass index (BMI), self-esteem and eating disorder psychopathology.

Measure	Total mean	Male Mean	Female mean	T statistic	P value
Age	28 years	29 years	26 years	1.38	.65
BMI	21.7	22.7	20.7	2.48	.71
Self-esteem	22.6	22.8	22.4	2.72	.258
EDE-Q	.53Δ	.48Δ	1.05Δ	−2.45‡	.013‡ *

ΔMedian.

‡Mann Whitney U statistic.

* Significant at p<.05.

Self-esteem was measured using the Rosenberg self-esteem scale [31].

Eating disorder psychopathology was measured using the Eating Disorder Examination Questionnaire 6.0 (EDE-Q).

To control for cultural differences, all participants were required to have lived in a ‘western’ country for at least 12 months [30]. Social economic status was measured with years of education as an index; all participants had a minimum of 12 years education (i.e finished high school). Self-esteem was measured using the Rosenberg self esteem scale [31].

Participants were screened for current psychiatric conditions using the Mini International Neuropsychiatric Interview screen [32] and ED psychopathology using the Eating Disorder Examination Questionnaire (EDE-Q) (see below). A global score of 2.8 was used as a clinical cut off for the EDE-Q [33] Females were found to have significantly higher levels of ED psychopathology than males (see Table 1).

#### Materials

The experimental set-up was identical to that used for experiment one without SCR. An additional task, perceptual judgments of hip size, was measured using a 100 cm ruler.

#### Measures

Body satisfaction was measured using the *Body Image States Scale (BISS)* and the *Figure Rating Scale (FRS)*. The BISS is a six-item scale designed to measure body satisfaction at a particular instance in time. The BISS correlates with other body satisfaction measures and demonstrates internal consistency and construct validity [34]. In the current study Cronbach’s alpha varied between .72 and .83 across all (pre and post for each body size) conditions. Higher BISS scores represent higher body satisfaction.

The *FRS* consists of nine (numbered) silhouetted bodies ranging from emaciated to obese. Participants select images that represent both their ideal and current body size. Scores (current – ideal) >0 indicate a desire to be thinner, scores <0 indicate a desire to be heavier and a score of 0 indicates satisfaction with body size. The FRS has good test-retest reliability [35].

ED psychopathology was measured using the *Eating Disorder Examination Questionnaire (EDE-Q) 6.0*, which has good consistency and reliability [36] and the current data had Cronbach’s alpha of .88. The questionnaire consists of 28-items rated on a seven-point Likert scale, except six items asking about frequency of behaviour. The questionnaire can be divided into four subscales (dietary restraint, eating concern, weight concern, and shape concern), or a single global measure. Because the global score was used to define clinical cut-off [33] it was this score that was used for all subsequent analysis. Additional measures were also taken to examine individual differences; details can be found in methods S1.

#### Procedure

The experiment utilised a within participants design. The full body illusion over wider (LB) and slimmer (SB) body types was performed as in experiment one. However, to prevent the experiment being too long and to limit the number of repetitions of the subjective questionnaires, only synchronous conditions were used. The first stroking (illusion) period was conducted in between giving pre and post-illusion BISS and FRS responses. Due to the small size manipulations in the illusion, the cognitive knowledge of being in an experiment, as well as other individual characteristics that may influence emotion, affective responses were hypothesized to be much smaller than any perceptual changes. Therefore, when measuring body satisfaction a longer duration of stroking was given (120 s), compared to when measuring perceptual responses, for the participants to be fully immersed in the illusion. Thus allowing more time not only for the perceptual recalibration, but also for the subsequent hypothesized affective response. In addition, the longer duration also provided a longer period between repetition of the BISS and FRS, reducing the likelihood of participants simply repeating remembered responses. The questions were delivered through the HMDs in a random order with the responses given verbally. To protect participant confidentiality the experimenter could not see the questions and reverse scoring was implemented.

Next, participants were asked to make perceptual judgments of their own hip size, for which the HMD screens were blank so that the participants had no vision of either their own body or that of the mannequin. Participants were required to hold a 100 cm ruler using the index finger and thumb of both hands with their arms outstretched in front of them. Participants then adjusted the distance between their index fingers to correspond with the perceived actual distance between their own hips. Whilst making the judgments participants could only move their right hand, with their left hand held in position by the experimenter preventing them from aligning their hands with sides of their body. For each trial, three separate judgments were made before and after each period of (60 s) stroking, bringing their hands to their sides between each judgment. This was repeated three times to gain sufficient power for reliable statistical analysis. It was made clear to participants that these judgments were to reflect perceived width of their own body and not that of the mannequin. Finally, after an additional 60 s of stroking, participants answered a modified version of the illusion questionnaire (see table S1). Each participant completed the entire procedure (120 s stroking with pre and post-illusion BISS and FRS responses, three trials of 60 s stroking each with three pre and three post illusion hip size judgments and 60 s of stroking followed by the ownership questionnaire) for both LB and SB conditions separated by 15-minute break. The order of conditions was counterbalanced across participants. The duration of the entire experiment was approximately 90 minutes including the 15-minute break in between body size conditions.

## Results

### Experiment 1

Experiment one established equally strong illusions of ownership with the large and small mannequins for both male and female participants. For the questionnaire data an illusion score was calculated by averaging across all three illusion questions. Similarly, a control score was calculated by averaging across the two control questions. The data were ordinal and not normally distributed (Shapiro-Wilk test) so were analysed using non-parametric Wilcoxon signed rank tests, for which effect size is indicated by the probability of superiority for dependent measures (*PS_dep_*).

Wilcoxon signed rank tests revealed a significant effect of synchrony in the LB condition for both sexes (Males: z = −2.83, p = .005, *PS_dep_* = .71; Females: z = −3.19, p = .001, *PS_dep_* = .89) with higher scores for synchronous stroking (Males: median  = 1.67; Females: median  = 2.0) compared to asynchronous (Males: median  = −.67; Females: median  = 1.33). The same was true of the SB condition (Males: z = −3.47, p = .001, *PS_dep_* = .87, synchronous median  = 1.67, asynchronous median  = −1.0; Females: z = −3.18, p = .001, *PS_dep_* = .87, synchronous median  = 2.67, asynchronous median  = −.67). No such effects were found in either condition with the control scores (maximum z =  −1.11, p = .266) (See Figure 2a and b). Wilcoxon signed ranks tests revealed no effect on illusion strength (synchronous conditions) of body size condition for either males (z = −1.612, p = .107) or females (z = −1.20, p = .234). Moreover, Mann Whitney U tests revealed no significant differences in illusion strength between males and females for either the LB (z = −.781, p = .425) or SB conditions (z = −.947, p = .344).

A further two questions were included in the questionnaire asking directly about the perceptual effects of the illusion: “my body felt fatter than usual” and “my body felt thinner than usual”. Analysis of these using Wilcoxon signed ranks found a non-significant trend in female participants to agree that they felt thinner during the synchronous SB condition (z = −.18, p = .072), but not in the LB condition (z = −.495, p = .620) or with male participants in either condition (SB: z = −.527, p = .598; LB z = −.397, p = .691). No significant effects were found for the question “my body felt fatter than usual” (maximum z = −1.03, p = .301).

The SCR data were not normally distributed (Shapiro-Wilk test), but being ratio data were transformed using natural log and entered into a 2×2×2 mixed ANOVA (after transformation the data met both normality and homogeneity assumptions with Shapiro-Wilk and Levene’s tests respectively). Effect size is indicated by partial eta squared (*np* ^2^). The within factors were Body Size (LB, SB) and Synchrony (Synchronous, Asynchronous), and the between factor was Sex (Male, Female). There was a significant main effect of Synchrony (f(1,25) = 5.72, p = .025, *np* ^2^ =  .19) with Synchronous conditions (mean  = .419) having greater SCR peaks compared to Asynchronous conditions (mean  = .339) (see Figure 2c). Neither the main effect of Body Size (f(1,25) = .001, p = .975) nor any interactions were significant (maximum = f(1,25) = 1.87, p = .184). The main effect of Sex was borderline significant (f(1,25) = 4.28, p = .05, *np* ^2^ = .15) with females having a slightly higher SCR ( mean  = .46) compared to males (mean  = .29). Although this may reflect a slight heightened response to seeing a knife for females, lack of a significant interaction suggests that this was not modulated by the illusion. Therefore, experiment one demonstrates equivalent illusion strength between males and females as well as for both body size conditions. These results mean that the paradigm is suitable to examine possible links to emotional experience (experiment two) as the conditions are matched in terms of vividness of the illusion.

### Experiment 2

After confirming the suitability of the paradigm, the second experiment then aimed to establish affective responses with pre and post-illusion body satisfaction measures. As found for experiment one Wilcoxon signed ranks tests revealed statistically equivalent illusion affirmation for the questionnaire scores for both body sizes (SB median  = 1.5; LB median  = 1.75; z = −1.63, p = .098). Man Whitney U tests also revealed equivalent scores for males and females in both LB (male median  = 1.5; female median  = 2.0; z = −.826, p = .418) and SB (male median  = 1.5, female median  = 2.0; z = −.904, p = .385) conditions.

Also included in the questionnaire were two questions asking directly about the perceptual effect of the illusion “my body felt thinner than usual” and “my body felt fatter than usual”. Wilcoxon signed ranks tests revealed no significant difference between responses to these questions for either condition (maximum z = −.617, p = .538). No significant differences were found between male and female responses for either question in either condition (z = −1.82, p = .075 uncorrected). For additional results see table S1 and results S1.

**Figure 3:**
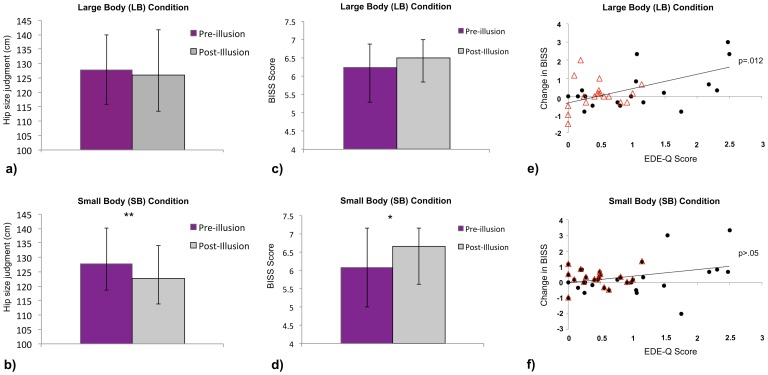
Results of Experiment two. Medians of pre and post-illusion hip judgments for the large (a) and small (b) body conditions Error bars show interquartile range. Medians of pre and post-illusion body satisfaction (BISS) scores for the large (c) and small (b) body conditions. Error bars show interquartile range. *  = p<.05, **  = p<.01 Scatter charts of change in body satisfaction (post – pre-illusion BISS scores) and eating disorder psychopathology (EDE-Q score) for large (e) and small (f) body conditions. Scores of male participants are depicted by open triangles and females by filled circles. A significant relationship was only found for the large body condition (p = .012).

The effects on perceived body size were measured using perceptual hip size judgments that were calculated as the distance between the inner edge of left and right index fingers on the ruler. This was then converted into a percentage of actual hip size for each participant in each condition with actual hip distance taken as the distance between the outer edges of the body at the hipbones. The data were normally distributed (Shapiro-Wilk test), but violated homogeneity assumptions, which was not rectified by transformation. Therefore the data were analysed using a nonparametric Friedman test assessing the factors Body Size (LB and SB) and Time (pre and post), effect size is indicated by Kendall’s W (*W^a^*) strength-of-relationship index and the probability of superiority for dependent measures (*PS_dep_*). The initial Friedman test was significant (÷2(3) = 12.16, P = .007, *W^a^*  = .11). Follow up Wilcoxon signed ranks tests conducted to directly test our predictions found no significant difference in the LB condition (z = −.72, p = .473). However, in line with our hypothesis, post-illusion judgments (mean  = 122.9%) were significantly smaller than pre-illusion judgments (mean  = 128.2%) in the SB condition (z = −4.47, p<.001, *PS_dep_* = .79) (see Figure 3d). Thus, illusory ownership of a slimmer body caused decreases in perceived (actual) body width. Sex differences were analysed using a change in hip judgment score described below.

The emotional effects of the illusion were calculated by comparing pre and post-illusion body satisfaction scores. These were calculated for each participant in each condition by taking a mean of the six BISS responses after reverse scoring. As the data were not normally distributed (Shapiro-Wilk test) and ordinal Wilcoxon signed rank tests were used. In the SB condition post-illusion (median  = 6.67) BISS scores were significantly greater than pre-illusion (median  = 6.01) scores (z = −2.04, p = .042, *PS_dep_* = .67) (see Figure 3b). Importantly, these results provide the first experimental evidence for a direct link between affective and perceptual body representations using multisensory illusions, demonstrating increases in body satisfaction following illusory ownership over a slimmer body. However, no significant difference was found for the LB condition (z = −1.09, p = .275) (see Figure 3a). No significant effects were found using FRS scores (maximum z = −.758, p = .448).

A change in BISS score was calculated by subtracting pre from post-illusion scores such that positive and negative values represented increases and decreases in body satisfaction respectively and did not represent an absolute change. Mann Whitney U tests revealed no significant differences between males and females for either LB (z = −.63, p = .528), or SB (z = −.67, p = .501) conditions. Moreover, Mann Whitney U tests also revealed no sex differences for change in hip size judgments (post – pre-illusion judgments) for either condition (maximum z = −.673, p = .506), thus supporting equivalent perceptual effects of the illusion between sexes, but not providing evidence for the hypothesised increase in affective response for females.

Spearman’s rho correlations revealed a significant positive relationship between EDE-Q score and change in body satisfaction in the LB condition (*r_s_* = .405, p = .012), such that the greater levels of ED psychopathology the more positive the change in body satisfaction (See Figure 3e), with lower EDE-Q scores being more strongly related to negative changes. The relationship between change in hip judgments (pre-post) and EDE-Q score approached significance in the LB condition (*r_s_* = −.295, p = .072, the higher the EDE-Q score the more negative the change in hip size judgment), however, change in BISS and change in hip judgments did not correlate with each other (*r_s_* = .006, p = .973). No significant relationships were found with EDE-Q, change in BISS, or change in hip judgments in the SB condition (maximum *r_s_* = −.268, p = .104). No significant correlations were found for any of the experimental variables with illusion strength for either LB or SB conditions (maximum *r_s_* = .163, p = .329). For additional results see table S2 and results S2. Further supplementary analysis was conducted to investigate the possible effects of condition order (results S3) and participant height (results S4) on each of the experimental measures.

## Discussion

The current study was the first to experimentally manipulate perceived body size using multisensory illusions demonstrating a direct link between perceptual and affective body representations. After determining that neither sex of the participant nor illusory body size affected illusion strength it was shown that ownership over a slimmer body significantly decreases perceived body width and increases body satisfaction.

Linking the body to emotional experience is not a new concept. For example, experiments investigating embodied cognition demonstrate that bodily posture and action not only express current emotional state but also facilitate it [37]. Our results extend these ideas, demonstrating that perception of our own body size can have a direct influence on emotional experience. Neural networks associated with body perception including posterior parietal [38] and premotor areas [39], have previously been investigated independently from networks involved with emotional experience that incorporate the insula and the anterior cingulate cortex [40]. Identification of emotional changes driven by altered body perception, however, suggests these networks to be functionally connected. Indeed, reduced grey matter volume in both networks has been found in ED patients [41]. Moreover, these areas are also found to be activated during tasks requiring body size comparison with media images using healthy controls [42].

The absence of asynchronous conditions in experiment two meant that a direct comparison of the effects of illusory ownership to a non-ownership control condition was not possible. However, viewing images of slim/ideal bodies of others has been consistently found to reduce body satisfaction in both females and males [43]–[45]. Such decreases are thought to be driven by mental comparisons between the seen ideal bodies and the observer’s own body [46]. Therefore, simply viewing an ideal body (without ownership) is more likely to elicit decreases in body satisfaction rather than the increases observed in the current study. However, these previous body comparison studies use images taken from a third person perspective and thus are not directly comparable to the current study, in which the mannequin body was seen from a first person perspective. Visual perspective is found to play a significant role for body ownership in multisensory body illusions [47], [48] and although it has not been investigated directly in terms of body satisfaction it is thought that third person perspective is more important due to social connotations (how the body is viewed by other people) [45], [49]. Therefore, based on current knowledge, it is unlikely that simply viewing an ideal body from a first person perspective would increase body satisfaction without ownership, although further investigation into the role of visual perspective in body satisfaction is required.

Decreases in hip size judgments are also unlikely to be explained purely by the size of the prior visual input of a slimmer body. Not only were participants explicitly instructed to make judgments of the perceived size of their own body and not that of the mannequin, but also our results are compatible with numerous previous studies, which have consistently demonstrated that multisensory illusions (and not just visual input of a distorted body size) can modulate size perception of own body parts [24], [25], [26], [28], [38].

Interestingly, the current data did not reveal sex differences in perceptual or emotional responses to the illusion. This supports previous findings of equivalent perceptual responses to multisensory illusions [20]. However, whereas this was only demonstrated previously using a male mannequin leaving the possibility of females having a stronger illusion with a female mannequin, the current study confirms similar illusion strength using sex-matched mannequins. Conversely, the current results do not support the notion of increased emotional responses for females. Because of the repetition of the BISS in a short time frame and the relatively small alteration of body size, the experiment may not be sensitive enough to tease apart subtle sex differences. However, much of the research to date has focused on body *dissatisfaction*, such that men are less likely to be dissatisfied with an overweight body (and more dissatisfied with an underweight body, e.g. [18]). Demonstrated here, however, was an *increase* in body *satisfaction*, such that whilst men may not be as disturbed by feeling fat, they may have equivalent positive effects of feeling slim.

Contrary to predictions, illusory ownership over a wider body did not reduce body satisfaction or increase perceptual body size. One reason for a lack of emotional response could be due to the mannequin physic, as even in the LB condition the mannequin had a flat stomach and muscle definition meaning that the experimental manipulation did not produce a true socially undesirable (fat) body type. This suggests that the observed emotional effects may not be caused by body size *per se* but that body shape is also important. This may also help to explain the lack of sex difference in emotional effects of the illusion. The socially ideal body shape for women is slim, whereas for males the ‘ideal body’ is both slim *and* muscular [50], [17], which, due to the muscle definition on the mannequin, was achieved by the SB condition. Thus the observed increases in body satisfaction may not be caused by a universal drive for slimness (feeling satisfied because you feel slimmer), but a drive for the social ideal (feeling satisfied because you perceive your body to resemble an ‘ideal’ body shape).

The absence of a significant effect of owning a larger body on perceptual judgments of hip size may reflect a difference in the brain’s propensity to expand or contract the neural representation of the body. Indeed, some studies investigating multisensory illusions have found asymmetries of illusion effects for larger and smaller limbs. However, contrary to the current findings, such studies tend to demonstrate stronger effects for larger body parts implying that body representations adapt more readily to increases rather than decreases in size [51]–[53]. Not all multisensory illusions appear to have asymmetric effects in regards to changes in body size, however, as equivalent perceptual effects have been found for illusions of owning giant and doll sized legs [29]. Thus it suggested that contractions of the body representation can occur easily as long as relative body proportions are maintained [29], [53]. In the current study, not only did both large and small body illusions maintain bodily proportions and elicit equivalently strong subjective experience, but also it was only the smaller body that produced a significant change in perceived hip size. Therefore, the current findings cannot be explained by the brain adapting more readily to expansions of the body. Alternatively our results may be explained by a general overestimation of actual body size. Individuals of low and normal weight (the majority of the current sample) tend to overestimate actual body size, whereas overweight individuals underestimate actual body size [12]. Thus the larger body in the LB condition, may not actually be perceived as larger by most participants, but instead as close to their normal perceived body size. Indeed, the average pre-illusion hip size judgment was greater than 100% demonstrating a default overestimation of body size. This, along with the mannequin physic, may also help to explain the lack of systematic affective response for the LB condition, as not all participants perceive the larger body to actually be larger than there own.

Responses to the Figure rating scale (FRS) were not significantly affected by the illusion in either body size condition. One reason for this may be that, because the wording of the question did not explicitly refer to how the body feels, participants were basing their judgments on cognitive knowledge of actual body size rather than a subjective feeling of body size [54]. Additionally, as the scale consists of only nine figures set out in size order, participants can easily remember which figure they selected previously [55] thus making cognitive based judgments more likely.

Feelings of body size were measured in the illusion questionnaire, as two questions were included that directly asked about whether the participant *felt* thinner or fatter than normal (see table S1). Responses to these questions did not, however, reveal any significant differences between conditions (although anecdotally some participants did spontaneously report explicit changes). This, together with the non-significant FRS results, may suggest that body satisfaction can be increased with only implicit perceptual (judgments of hip size) changes in body size. Future studies should therefore use greater manipulations of body size to examine the effect of more explicit perceptual changes on body satisfaction, which are likely to be more pronounced.

Although illusory ownership over a wider body did not have an overall effect on body satisfaction, increases in body satisfaction were found to correspond with higher ED psychopathology. Initially this finding seems counter-intuitive, as EDs are thought to correspond with greater sensitivity to body size and thus should be more dissatisfied with a larger body. However, such individuals also have a more negative and/or inaccurate perception of their actual body (i.e. perceive their real body size as larger). Therefore, although the mannequin in the LB condition did not conscribe to universal ideals as it did in the SB condition (and so did not increase body satisfaction universally across participants), it may be that the greater dissatisfaction an individual has towards their own body, the better (slimmer), this new mannequin body appears in comparison.

Furthermore, when viewing their own body, individuals with EDs pay more attention to areas associated with anxiety [56]. From first person perspective the stomach is the most salient of these body parts and, as such, may receive greater attention in those with higher ED psychopathology (more than overall body width). Therefore, for these individuals the feeling of having a flat stomach may become more important for body satisfaction than body width. Although attending to anxiety provoking body parts (body checking) tends to decrease body satisfaction even in healthy individuals [57], positive feedback from such checks can lead to a temporary feeling of being thin [14] and thus increase body satisfaction. The mannequin stomach is not only flat but also rigid (made from hard fibreglass) such that, as the experimenter strokes the torso this may provide positive feedback of a flat, smooth stomach, which is more important and heavily attended to by individuals with higher ED psychopathology.

Although these data do not answer the debate about body perception in ED they do add to our current knowledge demonstrating that changes in emotional response to a perceptual body illusion are related to ED psychopathology, even in a non-clinical sample. This finding is compatible with the idea of fluctuating body representations in ED [14] in respect to the affective body representation, but importantly demonstrating this with positive fluctuations (increases in body satisfaction) and thus may be more clinically relevant.

Demonstrating a positive affective response with the full body illusion that is modulated by ED psychopathology may suggest clinical applications for these methods. Some clinical treatments already incorporate virtual reality techniques to address anxiety-provoking environments [58] as well as body perception [59]. Moreover, multisensory illusions have already had some therapeutic success for other disorders thought to involve abnormalities in the cortical body representation (e.g. [60], [61]). Evidence for neural abnormalities related to body perception has also been found in EDs [40]. However, further research into underlying pathophysiology of EDs and the mechanisms underlying the affective responses to multisensory illusions, as well as longevity of any after-effects, is required before these methods can be considered for the clinic.

In sum, the current study provides the first experimental evidence for a direct link between body perception and body satisfaction using multisensory illusions. Moreover, non-clinical levels of ED psychopathology were related to affective responses to the illusion of owning a larger (wider) body suggesting a significant role of body perception in ED.

## Acknowledgments

The authors would like to thank Dr. Alexander Skoglund for technical support and writing the code for the experimental paradigms.

## Supporting Information

Table S1 Medians, z statistics and p values (uncorrected) for each questionnaire item used for experiment two. Questions 1 to 6 were also used for experiment one.
(DOCX)

Table S2 Correlation matrix of control variables. * = p<.05, ** = p<.001. EDE-Q = Eating disorder examination questionnaire; BMI = Body Mass Index. Control, shame, and Surveillance are subscales of the Objectified Body Consciousness Scale. Hip size = distance between the sides of participants’ body at the hipbones.
(DOCX)

Methods S1 Additional measures used for experiment two.
(DOCX)

Results S1 Additional analysis of questionnaire items for experiment two.
(DOCX)

Results S2 Correlational analysis for additional measures in experiment two.
(DOCX)

Results S3 Condition order analysis for experiment two.
(DOCX)

Results S4 The effect of participant height relative to the mannequin for experiment two.
(DOCX)
