# Supplementary material for: Illusory Changes in Body Size Modulate Body Satisfaction in a Way That Is Related to Non-Clinical Eating Disorder Psychopathology
Source: PLoS One. 2014 Jan 21;9(1):e85773. doi: 10.1371/journal.pone.0085773 (PMC3897512; doi:10.1371/journal.pone.0085773)
Supplement: Table S1 — Medians, z statistics and p values (uncorrected) for each questionnaire item used for experiment two. Questions 1 to 6 were also used for experiment one. (DOCX) [file pone.0085773.s001.docx]

| Number | Questionnaire item | SB median | LB median | Z statistic | p value |
| --- | --- | --- | --- | --- | --- |
| 1 | I seemed to feel the touch given to the mannequin | 2.0 | 2.0 | -1.37 | .171 |
| 2 | It felt like the mannequin body was my body | 1.0 | 1.0 | -1.09 | .277 |
| 3 | It felt as if I had two bodies | -2.0 | -2.0 | -.464 | .643 |
| 4 | It felt as though my body had turned into a plastic body | -1.5 | -2.0 | -1.15 | .249 |
| 5 | My body felt fatter than usual | -2.0 | -1.5 | -.617 | .538 |
| 6 | My body felt thinner than usual | 0.0 | 0.0 | -.088 | .930 |
| 7 | The mannequin body was attractive | 1.0 | 1.0 | -.734 | .463 |
| 8 | The mannequin body looked like my body | 1.0 | 1.0 | -1.41 | .158 |
