# Supplementary material for: Illusory Changes in Body Size Modulate Body Satisfaction in a Way That Is Related to Non-Clinical Eating Disorder Psychopathology
Source: PLoS One. 2014 Jan 21;9(1):e85773. doi: 10.1371/journal.pone.0085773 (PMC3897512; doi:10.1371/journal.pone.0085773)
Supplement: Table S2 — Correlation matrix of control variables. * = p<.05, ** = p<.001. EDE-Q = Eating disorder examination questionnaire; BMI = Body Mass Index. Control, shame, and Surveillance are subscales of the Objectified Body Consciousness Scale. Hip size = distance between the sides of participants’ body at the hipbones. (DOCX) [file pone.0085773.s002.docx]

|  | Measure | EDE-Q | Self-esteem | Control | Shame | Surveillance | Hip size | BMI |
| --- | --- | --- | --- | --- | --- | --- | --- | --- |
| EDE-Q | |  | *r_s_*=-.473**  p=.003 | *r_s_*=.001  p=.996 | *r_s_*=.542**  p=.000 | *r_s_*=.368*  p=.023 | *r_s_*=-.022  p=.895 | *r_s_*=.005  p=.976 |
| Self esteem | | *r_s_*=-.473**  p=.003 |  | *r_s_*=.235  p=.156 | *r_s_*=-.250  p=.130 | *r_s_*=-.239  p=.149 | *r_s_*=-.142  p=.369 | *r_s_*=.097  p=.561 |
| Control | | *r_s_*=.001  p=.996 | *r_s_*=.235  p=.156 |  | *r_s_*=-.072  p=.666 | *r_s_*=-.368*  p=.023 | *r_s_*=-.016  p=.922 | *r_s_*=.132  p=.431 |
| Shame | | *r_s_*=.542**  p=.000 | *r_s_*=-.250  p=.130 | *r_s_*=-.072  p=.666 |  | *r_s_*=.259  p=.117 | *r_s_*=-.064  p=.701 | *r_s_*=-.120  p=.473 |
| Surveillance | | *r_s_*=.368*  p=.023 | *r_s_*=-.239  p=.149 | *r_s_*=-.368*  p=.023 | *r_s_*=.259  p=.117 |  | *r_s_*=-.081  p=.630 | *r_s_*=-.249  p=.132 |
| Hip size | | *r_s_*=-.022  p=.895 | *r_s_*=-.142  p=.369 | *r_s_*=-.016  p=.922 | *r_s_*=-.064  p=.701 | *r_s_*=-.081  p=.630 |  | *r_s_*=.790**  p=.000 |
| BMI | | *r_s_*=.005  p=.976 | *r_s_*=.097  p=.561 | *r_s_*=.132  p=.431 | *r_s_*=-.120  p=.473 | *r_s_*=-.249  p=.132 | *r_s_*=.790**  p=.000 |  |
