# Supplementary material for: Illusory Changes in Body Size Modulate Body Satisfaction in a Way That Is Related to Non-Clinical Eating Disorder Psychopathology
Source: PLoS One. 2014 Jan 21;9(1):e85773. doi: 10.1371/journal.pone.0085773 (PMC3897512; doi:10.1371/journal.pone.0085773)
Supplement: Methods S1 — Additional measures used for experiment two. (DOCX) [file pone.0085773.s003.docx]

**Methods S1**

The *Mini International Neuropsychiatric Interview (MINI) Screen* is a 25-item diagnostic interview developed to quickly assess diagnoses of psychiatric patients; responses can then be followed up referring to the full MINI. The MINI has 85% agreement with expert diagnosis [32].  This interview was used as part of participant screening to ensure that all participants included in the sample did not have a current psychiatric condition.

The *Objectified Body Consciousness Scale (OBCS)* is a 24-item scale with three separate (8-item) subscales: body surveillance (viewing oneself as an observer), body shame (shame of not conforming to cultural standards), and appearance control beliefs (perceived control over body shape and appearance). All three subscales have good test re-test reliability [62].  This scale was used to measure different aspects of body consciousness not directly connected to ED psychopathology. Cronbach’s alpha for the current data demonstrated good reliability with each measure (body surveillance = .83, body shame = .74, appearance control beliefs = .74).

The *Rosenberg Self-Esteem Scale* is a 10-item, four-point Likert scale with responses ranging from “strongly agree” to “strongly disagree”. It has good reliability and validity [31]. Cronbach’s alpha for the current data was .87.

**Suplimentory reference:**

62. McKinley NM, Hyde JS (1996) The objectified body consciousness scale: Development and validation. Psychol Women Q 20: 181-215.
