# Supplementary material for: Illusory Changes in Body Size Modulate Body Satisfaction in a Way That Is Related to Non-Clinical Eating Disorder Psychopathology
Source: PLoS One. 2014 Jan 21;9(1):e85773. doi: 10.1371/journal.pone.0085773 (PMC3897512; doi:10.1371/journal.pone.0085773)
Supplement: Results S1 — Additional analysis of questionnaire items for experiment two. (DOCX) [file pone.0085773.s004.docx]

**Results S1:**

Analysis of each individual question revealed equal levels agreement in SB and LB conditions (see table S1). This was predicted for all statements except statements 5 (my body felt fatter than usual) and 6 (my body felt thinner than usual). Lack of significant effect for these items may be due to the magnitude of the body size manipulations, using adjustments of only +-15%, and the mannequin in the LB condition not conforming to a true socially undesirable (overweight) body type. In both conditions participants tended to disagree with the suggestion that they felt fatter than usual, consistent with the idea that neither mannequin appeared fat. When asked if they “felt thinner” median responses for both conditions were at zero, indicating uncertainty. Examining this statistically revealed that participants agreed more with question 6 (feeling thinner) compared to question 5 (feeling fatter) for both conditions (LB: z=-2.08, p=.037; SB: z=2.58, p=.01 uncorrected).

Spearman’s rho correlations found that agreement with statement 6 positively correlated with change in body satisfaction in the SB (*r_s_*=.390, p=.015) and LB (*r_s_*=.387, p=.016) conditions. Thus as participants felt thinner, their body satisfaction increased. Responses to statement 5 did not correlate with any of the experimental variables (maximum *r_s_*=-123, p=.463).

Responses to statement 7 (the mannequin body was attractive) correlated with change in body satisfaction in the SB condition (*r_s_*=.394, p=.014) such that greater agreement was associated with a greater increase in body satisfaction. The same relationship also approached significance in the LB condition (*r_s_*=.308, p=.060). This relates increases in body satisfaction to the appearance of the embodied mannequin. Agreement with statement 7 correlated with illusion score in the SB condition (*r_s_*=.424, p=.008) and approached significance in the LB condition (*r_s_*=.295, p=.072), such that greater agreement was associated with greater ownership. Statement 8 (The mannequin body looked like my body) also correlated positively with illusion score in both the SB (*r_s_*=.588, p<.001) and LB (*r_s_*=.541, p=.001) conditions; greater agreement associated with greater strength of ownership. This result fits with previous findings that strength of the Rubber hand Illusion positively correlates with perceived similarity to the real hand [63].

**Supplimentory reference**

63. Longo MR, Schüür F, Kammers MPM, Tsakiris M, Haggard P (1999) Self awareness and the body image. Acta Psychol 132: 166-172.
