# Supplementary material for: Illusory Changes in Body Size Modulate Body Satisfaction in a Way That Is Related to Non-Clinical Eating Disorder Psychopathology
Source: PLoS One. 2014 Jan 21;9(1):e85773. doi: 10.1371/journal.pone.0085773 (PMC3897512; doi:10.1371/journal.pone.0085773)
Supplement: Results S2 — Correlational analysis for additional measures in experiment two. (DOCX) [file pone.0085773.s005.docx]

**Results S2:**

Correlations between the different control variables were calculated to determine whether relationships that would be predicted by the literature were present in the current sample. EDE-Q score was found to correlate positively with the Shame and Surveillance subscales of the OBCS as well as with self-esteem, but not with BMI. The Shame and Surveillance OBCS subscales have previously been related to desired changes in weight and body size [63] as well as disordered eating [61] although unlike the EDE-Q such scales were not developed as a clinical assessment, which may explain why, unlike the EDE-Q, they do not correlate with fluctuations in body representation (changes in body satisfaction). This therefore may suggest that the observed deviations in body satisfaction are related to clinical ED opposed to body dissatisfaction *per se.* Similarly, self-esteem has frequently been related to EDs and body satisfaction (e.g. [64]) such that a correlation with EDE-Q responses was to be expected. This is also supported in the current sample. However, although self-esteem was found to correlate with EDE-Q it did not correlate with observed change in body satisfaction. Thus the current findings cannot be explained by self-esteem.

The appearance control beliefs OBCS subscale was found only to negatively correlate with the surveillance OBCS subscale, such that greater body surveillance is associated with less perceived control over appearance (body shape and weight). The appearance control beliefs subscale was originally developed to tap into one of the key aspects of body consciousness, proposing that it directly relates to both EDs and body dissatisfaction [61]. Subsequent research, however, suggests that this relationship is less consistent than for shame and surveillance [65], which is supported by the current data finding no significant correlations between appearance control beliefs and shame or EDE-Q. Nevertheless, some connection with body consciousness is supported as the data do suggest that those who feel more helpless in terms of their body’s appearance tend to pay more attention to it (see Table S2).

Body mass index (BMI) is calculated as participant body mass (weight) divided by the square of their height. Previously, BMI has been strongly associated with body dissatisfaction and disordered eating [15], which was not found in the current study. BMI is thought to contribute most strongly to body satisfaction for underweight males and obese males and females [17, 18]. The current study had no obese participants, with the vast majority falling within the healthy BMI range. Just three participants had a BMI that was above the normal healthy range (>25) and only two participants (both females) had recorded a BMI that would be considered underweight (<18). Therefore, lack of a significant relationship between EDE-Q and BMI may be due to the relatively low variance of BMI within the sample.

BMI was found to correlate strongly with hip size (distance between the outer edges of the body at the hipbones as measured by the experimenter). This finding supports the validity of the measure as an index of body size within the current experiments. For a full outline of these results please see Table S2.

**Supplimentory References:**

63. Forbes GB, Jobe RL, Revak JA (2006) Relationships between dissatisfaction with specific body characteristics and the Sociocultural Attitudes Toward Appearance Questionnaire-3 and Objectified Body Consciousness Scale. Body Image 3: 295–300.

64. van den Berg PA, Mond J, Eisenberg M, Ackard D, Neumark-Sztainer D (2010) The link between body dissatisfaction and self-esteem in adolescents: Similarities across gender, age, weight status, race/ethnicity, and socioeconomic status. J Adolesc Health 47: 290-296.

65. Knauss C, Paxton SJ, Alsaker FD (1998) Body Dissatisfaction in Adolescent Boys and Girls: Objectified Body Consciousness, Internalization of the Media Body Ideal and Perceived Pressure from Media. Sex Roles 59: 633-643.
