# Supplementary material for: Illusory Changes in Body Size Modulate Body Satisfaction in a Way That Is Related to Non-Clinical Eating Disorder Psychopathology
Source: PLoS One. 2014 Jan 21;9(1):e85773. doi: 10.1371/journal.pone.0085773 (PMC3897512; doi:10.1371/journal.pone.0085773)
Supplement: Results S3 — Condition order analysis for experiment two. (DOCX) [file pone.0085773.s006.docx]

**Results S3:**

All participants took part in both conditions, the order of which was counterbalanced between participants. Supplementary analysis was conducted to ensure that the order in which the conditions were completed did not affect the results. Participants who completed the LB condition first were compared to those who completed the SB condition first for all experimental variables. Non-normally distributed data was analysed with Mann Whitney U tests revealing no significant differences in change in body satisfaction for LB (z=-.353, p=.724) or SB (z=-.842, p=.40) conditions. Similarly, no significant effect of order was found for the illusion scores for either the LB (z=-.325, p=.745) or SB (z=-.282, p=.778) conditions. Independent t tests revealed no significant effect of order for either condition (LB: t(36)= -1.073, p=.29; SB: LB: t(36)= .588, p=.56) for hip size Judgments.
