# Supplementary material for: Illusory Changes in Body Size Modulate Body Satisfaction in a Way That Is Related to Non-Clinical Eating Disorder Psychopathology
Source: PLoS One. 2014 Jan 21;9(1):e85773. doi: 10.1371/journal.pone.0085773 (PMC3897512; doi:10.1371/journal.pone.0085773)
Supplement: Results S4 — The effect of participant height relative to the mannequin for experiment two. (DOCX) [file pone.0085773.s007.docx]

**Results S4:**

The different body size conditions were calculated relative to the actual width of each participant, but this calculation did account for variations in participant height. For participants who were taller than the mannequin, the mannequin hips would appear closer to the eyes through the HMDs, so that the body width may appear bigger. The opposite is true for those shorter than the mannequin; thus great deviations in height may exaggerate or depreciate the manipulations. After completing the experiment participant height was measured using a wall-mounted tape measure and then calculated as a proportion of mannequin height (PMH). Overall participant height was similar to that of the mannequin (mean PMH =.98): Males being slightly shorter than the male mannequin (mean=.94) and females slightly taller than the female mannequin (mean=1.02).

Because the data for proportion of mannequin height was normally distributed both parametric (Pearson’s *r*) and non-parametric (spearman’s rho) correlations were used for normally and non-normally distributed variables respectively. The relationship between PMH and change in body satisfaction for the SB condition was found to approach significance (*r_s_*=-.28, p=.089). The trend in the data indicates that the smaller PMH the greater the increase in body satisfaction. As stated above, for participants with a smaller PMH, the mannequin hips would appear smaller as they are further from the participant’s eyes. Therefore, the experimental manipulation in the SB condition would be exaggerated for these participants i.e. the slimmer mannequin would appear even slimmer with shorter participants. The fact that this relationship approaches significant for the SB condition with no similar trend in LB condition (*r_s_*=.033, p=.845) may act as further support that increases in body satisfaction in the SB condition are driven by the experimental manipulation (slimmer mannequin body). PMH did not correlate with any other variables in either condition (maximum *r*=-.114, p=.496).
